# Pharmacological Incongruity of Lacosamide in the Management of Delirium Complicated by Ileus

**DOI:** 10.1002/npr2.70131

**Published:** 2026-05-19

**Authors:** Takahiko Nagamine

**Affiliations:** ^1^ Sunlight Brain Research Center Hofu Yamaguchi Japan

**Keywords:** antiepileptic drugs, brain‐gut Axis, delirium, enteric nervous system, ileus, lacosamide

## Abstract

Delirium involves neurotransmitter imbalances and disrupted neural networks. Although lacosamide may theoretically treat delirium by reducing neuronal hyperexcitability via sodium channel modulation, clinical evidence is lacking. Sodium channels in the enteric nervous system mean lacosamide can cause gastrointestinal dysmotility independent of anticholinergic pathways. Given these risks and potential disruptions to the gut‐brain axis, off‐label use requires extreme caution and rigorous monitoring.

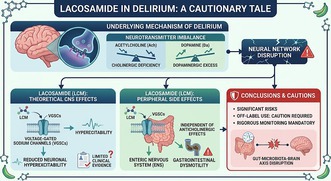

To the Editor,

The article by Sato on the efficacy of lacosamide (LCM) in four cases of delirium complicated by ileus was read with interest [1]. Although the author presents an intriguing alternative for managing agitation where antipsychotics are contraindicated, two major pharmacological issues require further scrutiny: the mismatch between LCM's mechanism and delirium pathology, and the potential for LCM to exacerbate gastrointestinal dysfunction.

## 1 Mechanistic Considerations in Delirium Pathophysiology

The primary concern pertains to the pharmacological rationale for using antiepileptic drugs (AEDs) like LCM in delirium. Although the pathophysiology of delirium is multifactorial, prominent hypotheses involve a “final common pathway” characterized by cholinergic deficits and dopaminergic hyperactivity [2]. Sato suggests that LCM may be beneficial via the modulation of voltage‐gated sodium channels (VGSCs), potentially attenuating NMDA‐mediated neurotoxicity. However, although this mechanism is relevant in epilepsy, its translation to the acute neurochemical imbalances of delirium remains speculative.

Unlike antipsychotics, LCM lacks direct affinity for dopamine or acetylcholine receptors. While stabilizing hyperexcitable membranes might provide symptomatic sedation, it does not address the underlying receptor‐level disturbances. Furthermore, pharmacovigilance data (e.g., FAERS) have linked third‐generation AEDs like LCM to paradoxical “forced normalization” or neuropsychiatric adverse events, including acute psychosis [3]. Indiscriminate reduction in neuronal excitability without addressing the underlying pathology may mask symptoms while potentially extending cognitive dysfunction.

## 2 Theoretical Risks in the Context of Paralytic Ileus

The second issue involves the appropriateness of LCM for patients with ileus. Sato posits that LCM is a safer choice due to its lack of an anticholinergic profile. However, the absence of anticholinergic activity does not render a drug inert regarding gastrointestinal motility. VGSCs are not exclusive to the central nervous system; they are vital components of the enteric nervous system (ENS) required for coordinating peristaltic reflexes. Mechanistically, the modulation of these channels by LCM has the potential to interfere with the signaling required for coordinated peristalsis. Although major clinical trials did not identify constipation as a primary adverse event, post‐marketing surveillance and real‐world databases have noted gastrointestinal complaints, including constipation, in some patient populations [4, 5].

Furthermore, the impact of AEDs on the “microbiota–gut–brain axis” is an emerging area of concern. Although direct studies on LCM in this context are currently limited, research indicates that various AEDs can alter gut flora composition, which may influence neuroinflammation and brain function via metabolic pathways [6]. In patients already suffering from paralytic ileus, the introduction of a sodium channel modulator might theoretically exacerbate intestinal stasis. Clinical priority in such cases should remain on systemic stabilization and restoring gastrointestinal function rather than introducing agents that could further complicate the primary pathology.

## 3 Precautions and Future Research

When utilizing AEDs off‐label for delirium, utmost caution is required. Clinicians must monitor for paradoxical agitation and metabolic changes, particularly in older patients. Pending high‐quality randomized controlled trials to evaluate whether LCM offers true therapeutic efficacy or merely temporary sedation, it is difficult to recommend its routine use for delirium complicated by ileus.

## Author Contributions

All aspects of this work were carried out by the sole author.

## Funding

The author has nothing to report.

## Consent

As this is a communication (review) article of existing literature, no direct interaction with patients or access to individual patient data occurred. This article adheres to ethical principles for academic research by accurately and fairly representing the information presented in the included studies. Proper citation and attribution are provided for all sources.

## Conflicts of Interest

The author declares no conflicts of interest.

## Data Availability

Data sharing not applicable to this article as no datasets were generated or analysed during the current study.
